# GH97 is a new family of glycoside hydrolases, which is related to the α-galactosidase superfamily

**DOI:** 10.1186/1471-2164-6-112

**Published:** 2005-08-30

**Authors:** Daniil G Naumoff

**Affiliations:** 1 Laboratory of Bioinformatics, State Institute for Genetics and Selection of Industrial Microorganisms, I-Dorozhny proezd, 1, Moscow 117545, Russia

## Abstract

**Background:**

As a rule, about 1% of genes in a given genome encode glycoside hydrolases and their homologues. On the basis of sequence similarity they have been grouped into more than ninety GH families during the last 15 years. The GH97 family has been established very recently and initially included only 18 bacterial proteins. However, the evolutionary relationship of the genes encoding proteins of this family remains unclear, as well as their distribution among main groups of the living organisms.

**Results:**

The extensive search of the current databases allowed us to double the number of GH97 family proteins. Five subfamilies were distinguished on the basis of pairwise sequence comparison and phylogenetic analysis. Iterative sequence analysis revealed the relationship of the GH97 family with the GH27, GH31, and GH36 families of glycosidases, which belong to the α-galactosidase superfamily, as well as a more distant relationship with some other glycosidase families (GH13 and GH20).

**Conclusion:**

The results of this study show an unexpected sequence similarity of GH97 family proteins with glycoside hydrolases from several other families, that have (β/α)_8_-barrel fold of the catalytic domain and a retaining mechanism of the glycoside bond hydrolysis. These data suggest a common evolutionary origin of glycosidases representing different families and clans.

## Background

On the basis of sequence similarity, glycoside hydrolases (or glycosidases, EC3.2.1.-) have been grouped into 96 families (GH1-GH100, except GH21, GH40, GH41, and GH60) by the Carbohydrate-Active Enzymes (CAZy) classification [1,2]. In the case of poly-domain proteins each catalytic domain is considered separately. A family was initially defined as a group of at least two sequences displaying significant amino acid similarity and with no significant similarity with other families [1]. Later, some related families of glycosidases have been combined into clans [3,4]. According to its definition, a clan is a group of families that are thought to have a common ancestry and are recognized by significant similarities in tertiary structure together with conservation of the catalytic residues and a catalytic mechanism [3]. Glycosidases catalyze hydrolysis of the glycosidic bond of their substrates via two general mechanisms, leading to either inversion or overall retention of the anomeric configuration at the cleavage point [4-6]. Currently, 14 clans (GH-A-GH-N) are described, and in total they contain 46 families [2]. Families of four clans (GH-A, GH-D, GH-H, and GH-K), as well as several other families, that have not been assigned to any clan, contain proteins with a similar (β/α)_8_-barrel fold of the catalytic domain [2]. Several glycosidases, that do not have any homologues, are included into a group of non-classified glycoside hydrolases [1,2]. In several instances, proteins from this group have been reclassified into new families when their homologues were found [7].

Two different clans have never been merged in the CAZy classification [2], even after their significant similarity has been established. Instead, related clans (and families) having statistically significant sequence similarity of the corresponding proteins were proposed to be grouped into superfamilies at a higher hierarchical level. For example, we have described the furanosidase (β-fructosidase) superfamily, that includes clans GH-F (inverting glycosidases) and GH-J (retaining glycosidases), as well as the GHLP (COG2152) family of enzymatically-uncharacterized proteins [8-11].

Nowadays, some families are very large. For example, GH13 family (clan GH-H) includes more than 2,000 representatives [2]. This large and poly-specific group of enzymes has been studied by many authors [12-19]. In particular, it was shown that splitting of this family into smaller subfamilies allowed to clarify the relationship of its members [12,13].

The majority of known glycosidases with the α-galactosidase activity [EC3.2.1.22] belong to families GH27 and GH36, that form clan GH-D [2,20]. This clan and family GH31 compose the α-galactosidase superfamily [21-24]. This superfamily has a distant relationship with clan GH-H [25,26], which we have proposed to name the α-glucosidase superfamily [24]. Both superfamilies contain proteins sharing the same enzymatic mechanism (retention), a similar (β/α)_8_-barrel fold of the catalytic domain [2], and use substrates only with the axial orientation of the glycosidic bond [4].

**Figure 1:**
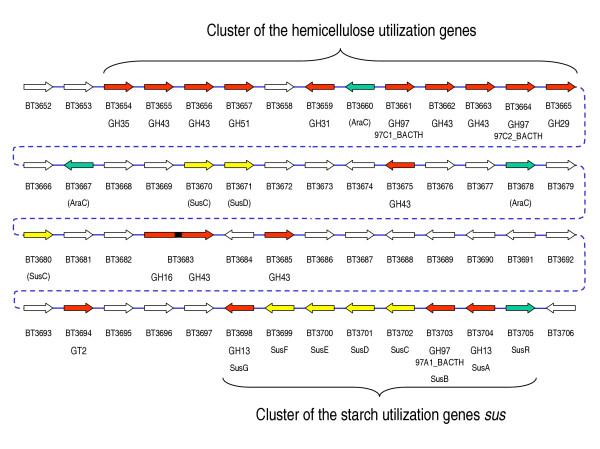
**Structure of *Bacteroides thetaiotaomicron *ATCC29148 genome fragment containing gene clusters for starch and hemicellulose utilization**. Arrows indicate the direction of gene transcription. Red arrows correspond to glycosidase (GH) and glycosyltransferase (GT) genes: family belonging is indicated. Yellow arrows correspond to genes coding outer membrane proteins involved in starch binding (*susC-susF*) and their homologues. Green arrows correspond to genes of the transcriptional activator SusR and predicted transcriptional regulators homologous to AraC.

Gram-negative obligate anaerobe *Bacteroides thetaiotaomicron *ATCC29148 is a commensal bacterium found in the human colon where it ferments a wide variety of polysaccharides [27,28]. Its starch utilization system (sus) has been studied in detail [29-35]. One of the corresponding loci (Figure 1) includes divergently oriented regulatory gene *susR *and seven structural genes *susA-susG *[30-34]. Genes *susC-susF *encode outer membrane proteins are involved in starch binding. Glycosidases SusA (a neopullulanase, EC 3.2.1.135) and SusG (an α-amylase, EC 3.2.1.1) are members of family GH13 [29-32]. SusB is an unusual α-glucosidase [EC 3.2.1.20] that for a long time was considered a unique glycosidase with no homologues [29,30]. Therefore it was included in the group of non-classified glycoside hydrolases [2]. We have found a group of its homologues among hypothetical proteins encoded by open reading frames (ORFs), that recently were sequenced in the frame of several prokaryotic genome projects. We referred to this group of proteins as the GHX family [23,24]. In June 2004, 18 members of this family were recognized in the CAZy classification as the GH97 family of glycoside hydrolases. Currently (June 2005), family GH97 includes two α-glucosidases SusB from closely related bacteria *B. thetaiotaomicron *ATCC29148 and *Tannerella forsythensis *(*Bacteroides forsythus*) ATCC43037, as well as 22 hypothetical proteins encoded by ORFs [2].

In this work we updated the GH97 family of glycosidases, performed its phylogenetic analysis, and established its evolutionary relationship with several other glycosidase families.

## Results and discussion

### Collecting sequences of family GH97

PSI-BLAST search of the non-redundant database with the *Bacteroides thetaiotaomicron *α-glucosidase SusB (97A1_BACTH, see Table I) as a query sequence yielded 32 protein sequences with the worst (the largest) *E*-value of 2 × 10^-20 ^during the first round. Among them we found 10 paralogous proteins from *B. thetaiotaomicron *ATCC29148 and their 22 homologues from other species. Among 32 obtained proteins were found all 24 members of the GH97 family listed at the CAZy server [2]. Genomic BLAST revealed 13 additional homologous sequences. Based on the sequence similarity, we propose to enlarge the GH97 family by including all known homologues of SusB. As a result, currently this family includes 45 proteins. The majority of them represent Eubacteria (16 different species). Three other sequences correspond to Archaea (*Haloarcula marismortui*) and two uncultured bacteria. Four sequences are annotated in the NCBI database as eukaryotic (*Anopheles gambiae*) genome fragments. Only five out of 45 protein sequences (from *Anopheles *and an uncultured bacterium) are short fragments (Table I).

**Table I:** Glycoside hydrolases analyzed in the work

Name	Family, subfamily	Organism	Accession number^a^	Protein function (annotation)	Length^b^
97A1_BACTH	GH97, 97a	*Bacteroides thetaiotaomicron *VPI-5482 = ATCC29148	AAC44671	alpha-glucosidase SusB	738
97A2_BACTH	GH97, 97a	*Bacteroides thetaiotaomicron *VPI-5482 = ATCC29148	AAO79686	ORF: alpha-glucosidase	719
97A3_BACTH	GH97, 97a	*Bacteroides thetaiotaomicron *VPI-5482 = ATCC29148	AAO75790	ORF: alpha-glucosidase	671
97B1_BACTH	GH97, 97b	*Bacteroides thetaiotaomicron *VPI-5482 = ATCC29148	AAO76978	ORF: putative alpha-glucosidase	662
97B2_BACTH	GH97, 97b	*Bacteroides thetaiotaomicron *VPI-5482 = ATCC29148	AAO78400	ORF: putative alpha-glucosidase	650
97B3_BACTH	GH97, 97b	*Bacteroides thetaiotaomicron *VPI-5482 = ATCC29148	AAO77727	ORF: alpha-glucosidase	649
97B4_BACTH	GH97, 97b	*Bacteroides thetaiotaomicron *VPI-5482 = ATCC29148	AAO78269	ORF: putative alpha-glucosidase	674
97C1_BACTH	GH97, 97c	*Bacteroides thetaiotaomicron *VPI-5482 = ATCC29148	AAO78766	ORF: alpha-glucosidase	647
97C2_BACTH	GH97, 97c	*Bacteroides thetaiotaomicron *VPI-5482 = ATCC29148	AAO78769	ORF: putative alpha-glucosidase	638
97E1_BACTH	GH97, 97e	*Bacteroides thetaiotaomicron *VPI-5482 = ATCC29148	AAO75239	ORF: putative alpha-glucosidase	644
97A1_BACFR	GH97, 97a	*Bacteroides fragilis *YCH46	BAD47941	ORF: alpha-glucosidase	719
97A2_BACFR	GH97, 97a	*Bacteroides fragilis *YCH46	BAD48072	ORF: alpha-glucosidase	671
97B1_BACFR	GH97, 97b	*Bacteroides fragilis *YCH46	BAD50730	ORF: putative alpha-glucosidase	649
97B2_BACFR	GH97, 97b	*Bacteroides fragilis *YCH46	BAD50235	ORF: putative alpha-glucosidase	649
97A1_TANFO	GH97, 97a	*Tannerella forsythensis *(*Bacteroides forsythus*) ATCC43037	AAO33827	alpha-D-glucosidase SusB	708
97A1_PREIN	GH97, 97a	*Prevotella intermedia *17	(TIGR_246198)	ORF	733
97A1_PRERU	GH97, 97a	*Prevotella ruminicola *23	(TIGR_264731)	ORF	737
97B1_PRERU	GH97, 97b	*Prevotella ruminicola *23	(TIGR_264731)	ORF	645
97B2_PRERU	GH97, 97b	*Prevotella ruminicola *23	(TIGR_264731)	ORF	658
97C1_PRERU	GH97, 97c	*Prevotella ruminicola *23	(TIGR_264731)	ORF	621
97C2_PRERU	GH97, 97c	*Prevotella ruminicola *23	(TIGR_264731)	ORF	639
97C3_PRERU	GH97, 97c	*Prevotella ruminicola *23	(TIGR_264731)	ORF	645
97A1_SALRU	GH97, 97a	*Salinibacter ruber *DSM13855	(NC_006812)	ORF	708
97A1_AZOVI	GH97, 97a	*Azotobacter vinelandii *AvOP	EAM07225	ORF: alpha-glucosidase	673
97A1_XANAX	GH97, 97a	*Xanthomonas axonopodis *pv. citri 306	AAM37448	ORF: alpha-glucosidase	693
97D1_XANAX	GH97, 97d	*Xanthomonas axonopodis *pv. citri 306	AAM38156	ORF: alpha-glucosidase	654
97A1_XANCA	GH97, 97a	*Xanthomonas campestris *pv. campestris ATCC33913	AAM41744	ORF: alpha-glucosidase	692
97D1_XANCA	GH97, 97d	*Xanthomonas campestris *pv. campestris ATCC33913	AAM42433	ORF: alpha-glucosidase	654
97A1_MICDE	GH97, 97a	*Microbulbifer *(*Saccharophagus*) *degradans *2–40	ZP_00315606	ORF: hypothetical protein	684
97B1_MICDE	GH97, 97b	*Microbulbifer *(*Saccharophagus*) *degradans *2–40	ZP_00317369	ORF: hypothetical protein	679
97C1_MICDE	GH97, 97c	*Microbulbifer *(*Saccharophagus*) *degradans *2–40	ZP_00317507	ORF: hypothetical protein	674
97C2_MICDE	GH97, 97c	*Microbulbifer *(*Saccharophagus*) *degradans *2–40	ZP_00315142	ORF: hypothetical protein	661
97A1_SHEON	GH97, 97a	*Shewanella oneidensis *MR-1	AAN55484	ORF: alpha-glucosidase	699
97A1_SHEBA	GH97, 97a	*Shewanella baltica *OS155	EAN43632	ORF: alpha-glucosidase	710
97A1_SHEFR	GH97, 97a	*Shewanella frigidimarina *NCIMB400	EAN73178	ORF: alpha-glucosidase	697
97A1_SHEDE	GH97, 97a	*Shewanella denitrificans *OS-217	EAN70289	ORF: alpha-glucosidase	727
97A1_SHEAM	GH97, 97a	*Shewanella amazonensis *SB2B	EAN38820	ORF: alpha-glucosidase	676
97A1_NOVAR	GH97, 97a	*Novosphingobium aromaticivorans *DSM12444	ZP_00303588	ORF: transketolase	682
97A1_SPHAL	GH97, 97a	*Sphingopyxis alaskensis *RB2256	EAN45679	ORF: alpha-glucosidase	680
97D1_CAUCR	GH97, 97d	*Caulobacter crescentus *CB15	AAK22781	ORF: putative alpha-glucosidase	670
97A1_ERYLI	GH97, 97a	*Erythrobacter litoralis *HTCC2594	EAL74063	ORF: alpha-glucosidase	681
97E1_RHOBA	GH97, 97e	*Rhodopirellula baltica *SH1 (*Pirellula *sp. 1)	CAD78916	ORF: alpha-glucosidase	645
97C1_LEIXY	GH97, 97c	*Leifsonia xyli *subsp. xyli CTCB07	(NC_006087)*	ORF: similar to alpha-glucosidase	775*
97X1_SOLUS	GH97	*Solibacter usitatus *Ellin6076	EAM58489	ORF: hypothetical protein	619
97A1_HALMA	GH97, 97a	*Haloarcula marismortui *ATCC43049	AAV45265	ORF: alpha-glucosidase	1144
97A1_ANOGA	GH97, 97a	*Anopheles gambiae *str. PEST (African malaria mosquito)	(AAAB01006165)	ORF	380*
97A2_ANOGA	GH97, 97a	*Anopheles gambiae *str. PEST (African malaria mosquito)	(AAAB01064948)	ORF	209*
97A3_ANOGA	GH97, 97a	*Anopheles gambiae *str. PEST (African malaria mosquito)	(AAAB01020110)	ORF	231*
97A4_ANOGA	GH97, 97a	*Anopheles gambiae *str. PEST (African malaria mosquito)	(AAAB01068263)	ORF	229*
97A1_UNBAC	GH97, 97a	uncultured murine large bowel bacterium BAC31B	AAX16382	ORF: alpha-glucosidase	720
97A2_UNBAC	GH97, 97a	uncultured bacterium	(AY350337)	ORF	106*
97A1_ENSEQ	GH97, 97a	environmental sequence (cf. *Shewanella *SAR-1)	EAJ06144*	ORF: unknown	703
97A2_ENSEQ	GH97, 97a	environmental sequence (cf. *Shewanella *SAR-2)	EAI69763	ORF: unknown	699
97A3_ENSEQ	GH97, 97a	environmental sequence	EAJ75652	ORF: unknown	714
97A4_ENSEQ	GH97, 97a	environmental sequence	EAI51202	ORF: unknown	713
97A5_ENSEQ	GH97, 97a	environmental sequence	EAI80962	ORF: unknown	702*
97A6_ENSEQ	GH97, 97a	environmental sequence	EAH92811, EAI03708, EAD44407, EAG79875, EAH92819, EAI36772	ORF: unknown	711
97A7_ENSEQ	GH97, 97a	environmental sequence	EAJ99185, EAD99255, EAH48404, EAH57728, EAD83763, EAH04981, EAC91563, EAH85977, EAD11728	ORF: unknown	710
97A8_ENSEQ	GH97, 97a	environmental sequence	EAJ85380, EAH86891	ORF: unknown	669*
97C1_ENSEQ	GH97, 97c	environmental sequence	EAD85224*	ORF: unknown	218*
GH27_ORYSA	GH27, 27a	*Oryza sativa *japonica cultivar Nipponbare (rice)	BAB12570	alpha-galactosidase	417
GH36_LACPL	GH36, 36A	*Lactobacillus plantarum *ATCC8014	AAF02774	alpha-galactosidase MelA	738
GH31_ECOLI	GH31	*Escherichia coli *K12	AAC76680	alpha-xylosidase YicI	772

^a^Accession numbers of protein sequences are given according to the NCBI database [72]. Numbers of nucleic sequences are given (in parentheses) if the corresponding protein sequences have not been deposited. In some cases (asterisked), protein sequences were edited by changing the start codon.

^b^Protein length was established as the number of amino acids in the corresponding precursor. Incomplete sequences (protein fragments) are asterisked.

**Figure 2:**
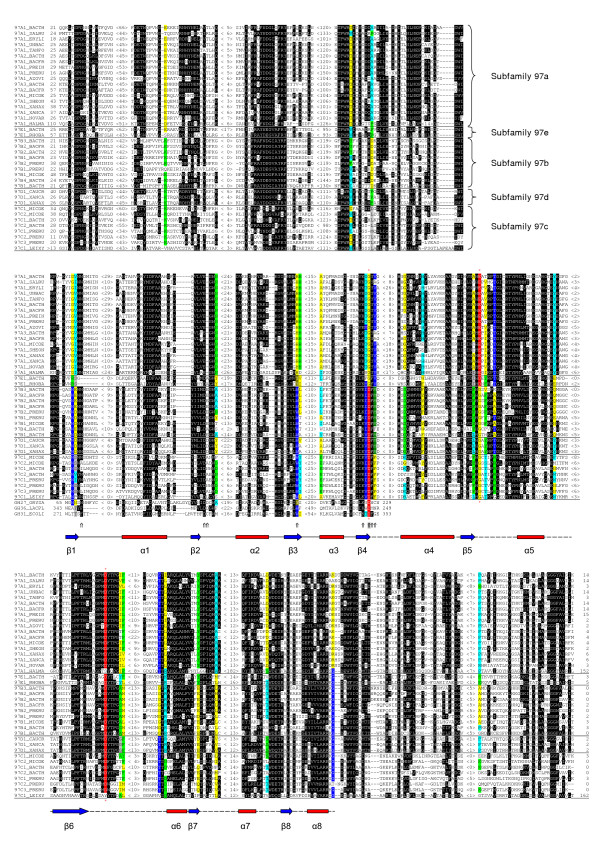
**Portion of the multiple sequence alignment of the sequences analyzed**. Ten-letter name for each sequence is indicated in the leftmost column (for origin of the sequences see Table I). The alignment continuously spans three panels. Distances to the N- and C-termini and length of omitted fragments are indicated. Highly conserved residues are highlighted in sequences. Amino acid positions that are highly conserved within several subfamilies but varied in amino acid residues in different subfamilies are coloured. Subfamily belonging of sequences (for family GH97) are indicated in the most right. Amino acid residues, interacting with the substrate in the active center of GH27 and GH31 family glycosidases, are indicated by arrows at the bottom [50-54]. The arrow on the gray background corresponds to the Asp residue, playing the role of the nucleophile in glycosidases of families GH27 and GH31. Red asterisks over and under the alignment indicate three conserved positions (in red) probably corresponding to the nucleophile and proton donor in the glycosidases of family GH97 (see text). Alignment of GH27_ORYSA and GH31_ECOLI is structure-based. At the bottom of the figure, β-strands and α-helixes of the (β/α)_8_-barrel are indicated. The first part of the barrel (β1–β4) is shown according to the known structures of GH27 and GH31 family members [51, 54]. The second part of the barrel (α4–α8) is based on generalization of predictions for several GH97 family proteins by 3D-PSSM, GOR IV, and nnpredict programs.

PSI-BLAST searches with a few randomly selected divergent representatives of the GH97 family used as a query sequence during the first round always yielded the same 32 protein sequences as with 97A1_BACTH. An analysis of the order of the sequence appearance during the first round of searches by PSI-BLAST, depending on the query, allows us to distinguish five subfamilies (97a–97e) in the GH97 family with at least two known members in each of them (Table I). The obtained pairwise alignments were used for generating the protein multiple sequence alignment of family GH97. The most conserved parts of the alignment are shown on Figure 2.

The fragment of *Leifsonia xyli *CTCB07 genome [GenBank: NC_006087] revealed by Genomic BLAST has 2 stop codons in the region homologous to genes of GH97 family proteins. An analysis of the nucleic acid sequence allowed us to detect a frame shift (data not shown). The improved ORF encodes protein sequence (97C1_LEIXY), showing a significant sequence similarity with the other members of family GH97 along its whole length (Figure 2). However, it was impossible to determine the very beginning of the protein sequence including the start codon. This protein is a divergent representative of the GH97 family and it could not be classified into any subfamily on the basis of pairwise sequence comparison. 97C1_LEIXY and its closest homologue 97D1_CAUCR (*E*-value = 2 × 10^-54^) have only 30% of sequence identity.

A short gene fragment [GenBank: AY350337] from an uncultured bacterium was revealed by Genomic BLAST. It had been obtained and sequenced during PCR screening of human gut microflora [36]. The deduced protein sequence (97A2_UNBAC) corresponds to the C-terminal part of the others GH97 family proteins and has the highest similarity level with 97A1_BACTH (63% of sequence identity) and 97A1_TANFO (60%). It allows us to include this protein fragment into subfamily 97a (Table I).

PSI-BLAST search of the non-redundant protein database yielded a unique eukaryotic protein fragment [GenPept: EAL42226] homologous to GH97 family proteins. Screening of the database of eukaryotic nucleic acid sequences uncovered the corresponding DNA sequence [GenBank: AAAB01006165], as well as three other short sequences [GenBank: AAAB01064948, AAAB01020110, and AAAB01068263]. All of them had been sequenced during the mosquito *Anopheles gambiae *genome project [37]. These 4 sequences were aligned for the identification of overlapping regions. AAAB01064948 sequence is homologous to the central part of AAAB01006165 sequence having 54% of identity at the protein level. The ends of AAAB01020110 sequence are respectively homologous to one end of AAAB01006165 and AAAB01068263 sequences: 65% and 69% sequence identity at the protein level. Thus, these 4 sequences correspond to at least two different genes. In total, they cover a complete bacterial gene encoding of a protein of family GH97. Taking into account i) a high similarity level of the 4 deduced protein sequences with bacterial proteins (50–71% identity with 97A1_BACFR, 97A2_BACTH, 97A1_TANFO, and 97A1_BACTH), ii) the intron-free gene structure, iii) an inability to map the genes on the mosquito chromosomes, and iv) absence of GH97 family proteins in any other eukaryotic organism, we suggest the bacterial origin of these four gene fragments. The bacterial origin could have resulted from a contamination of *Anopheles gambiae *tissue used for preparing of genome library by mosquito *Bacteroides*-like gut microflora. The evidence for such kind of contamination was obtained when testing the 35,575 clones from *A. gambiae *cDNA library [38]. It was found that at least 808 sequences appeared to be bacterial contaminants.

In order to enlarge database of family GH97 we performed screening of the so-called "Environmental Samples data" [39]. It revealed 60 nucleic acid sequences from the Sargasso Sea that are homologous to genes of GH97 family proteins. However, the majority of them encode only short protein fragments and many of them have a very high level of sequence similarity. Among them we found only 5 full-size or almost complete genes (each encodes a protein consisting of more than 650 amino acid residues). Three additional "gene" sequences were obtained by combining overlapping gene fragments with almost identical sequences (at least 95% of sequence identity at the protein level). Hypothetical proteins (97A1_ENSEQ-97A8_ENSEQ) encoded by these 8 genes should be placed in the 97a subfamily, on the basis of sequence similarity (Table I). Moreover, the majority of the incomplete genes encode protein fragments belonging to the same subfamily. Only four [GenPept: EAE76000, EAE67019, EAH16525, and EAH96685] and two [GenPept: EAE21375 and EAG68085] protein fragments correspond to subfamilies 97b and 97c, respectively. One short fragment (137 amino acids; [GenPept: EAD85224]) cannot be unambiguously classified into any subfamily of the GH97 family. An analysis of the nucleic acid sequence encoding the latter protein fragment [GenBank: AACY01501371] allowed us to extend the protein fragment by using another start codon. The resulting protein sequence (97C1_ENSEQ; 218 amino acids) shows similarity with the sequences of the other members of family GH97 along its whole length. However, it was still impossible to include this protein fragment into any subfamily on the basis of pairwise sequence comparison.

### Phylogenetic analysis of family GH97

To check the actual relationships of proteins within the GH97 family we performed a phylogenetic analysis using the obtained multiple sequence alignment. It is well known that phylogeny is the best basis for verification of subfamily structure of a protein family. In many works, where composition of a glycosidase family has been analyzed, the monophyletic status was used as the main argument for a subfamily description. Among others [40-44], this method has been applied to GH13 [12,13], GH27 [23,24], and GH36 [24] families of glycoside hydrolases.

**Figure 3:**
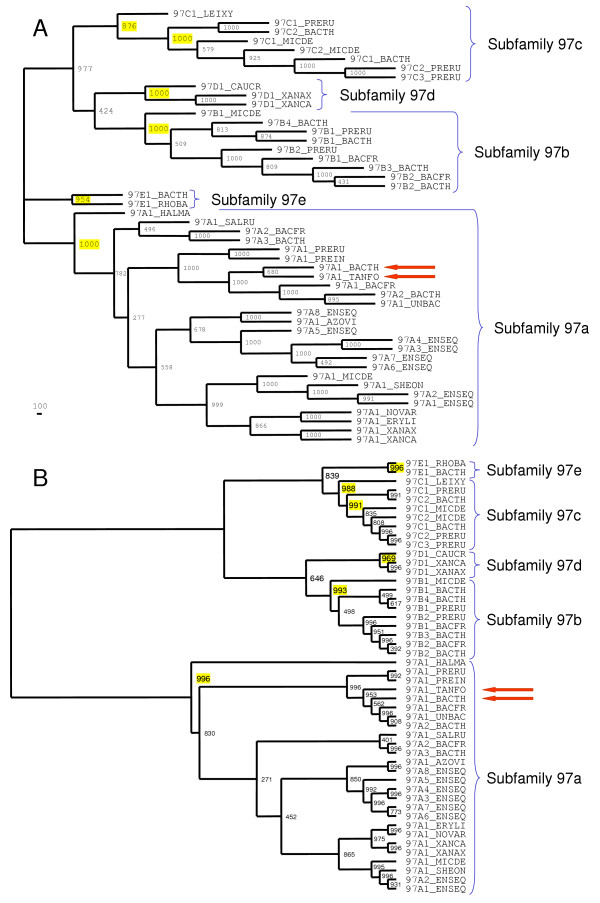
**Phylogenetic trees of family GH97**. The trees were reconstructed by the PHYLIP package. Each node was tested using the bootstrap approach and the number of supporting pseudoiterations (out of 1000) is indicated for each internal knot. Subfamily belongings of sequences are indicated, the value of bootstrap support for each subfamily is coloured in yellow. Red arrows indicate to the enzymatically-characterized proteins 97A1_BACTH and 97A1_TANFO (see text). The origin of sequences is given in Table I. (A) The maximum parsimony phylogenetic tree. The bootstrap values were determined using the maximum parsimony (PROTPARS) method. (B) The neighbor-joining phylogenetic tree. The number of amino acid substitutions per site is taken as a measure of branch length.

In order to verify our subdivision of the GH97 family into subfamilies we checked the clustering of the family members in the phylogenetic tree. The maximum parsimony (MP; Figure 3A) and the neighbor-joining (NJ; Figure 3B) trees have very similar topology, suggesting the correct interpretation of the evolutionary events. When any subfamily of the GH97 family was considered as an outgroup, both MP and NJ trees showed that all other subfamilies appear to form monophyletic groups with a high bootstrap value (at least 95.4% of support at both trees). It should be noted that there is no pair of subfamilies that compose neighbor clusters on both trees with significant bootstrap support. This suggests approximately the same evolutionary distance between each pair of the subfamilies.

The archaeal protein 97A1_HALMA is a clear outlayer in the cluster of subfamily 97a at MP and NJ trees (Figure 3). The other members of this subfamily compose several subclusters, that include representatives either from Bacteroidetes or Proteobacteria phyla.

Unclassified protein 97C1_LEIXY is the closest neighbor of subfamily 97c cluster at MP and NJ trees (Figure 3) and therefore it can be considered as a divergent representative of this subfamily (Table I). Phylogenetic analysis of 97C1_ENSEQ protein fragment (data not shown) allowed us to place it into the same subfamily 97c.

An analysis of the GH97 family multiple sequence alignment revealed a number of amino acid positions that are highly conserved within several subfamilies but varied in amino acid residues in different subfamilies (Figure 2). Taken together, these signature sequence positions allow to predict the subfamily belonging of a protein sequence.

### Relationship of family GH97 with some other glycosidase families

Depending on the GH97 query and the statistical significance threshold of *E*-value, during the second or third PSI-BLAST iterations, as a rule, we detected statistically significant similarities with α-galactosidases. They represent families GH27 and GH36 of clan GH-D (the α-galactosidase superfamily). More distant similarities were found with glycosidases of family GH31 (the α-galactosidase superfamily) and in some cases with enzymatically-uncharacterized proteins from COG0535. COG0535 has been annotated as a family of predicted Fe-S oxidoreductases, like the closest COG0641 [45]. Our BLAST searches show, that both COG families are related to the radical SAM superfamily of Fe-S enzymes [46], having (β/α)_8_-barrel fold [PDB: 1R30].

When we used some representatives of subfamily 97a (for example, 97A1_BACTH) as a query and an *E*-value cut-off of 0.01, it was possible to reveal statistically significant similarity with glycosidases of family GH20 (clan GH-K). A similarity with proteins of this family was detected after the second PSI-BLAST iteration, while the next one or two iterations revealed a distant relationship with members of COG0296 (family GH13 of clan GH-H). It should be noted that glycosidases from the clans GH-D, GH-H, and GH-K have a similar (β/α)_8_-barrel fold of their catalytic domain and the same molecular mechanism of the hydrolyzing reaction [2]. Thus, our results agree with the data of several authors [20,25,47-49] showing the relationship of glycosidases from GH13, GH27, GH31, and GH36 families. More detail analysis of these families and their relationship was done by Rigden [26].

Using the α-galactosidases from rice (GH27_ORYSA, family GH27) and *Lactobacillus plantarum *(GH36_LACPL, family GH36) as a query sequence for PSI-BLAST searches we found their homology with some representatives of the GH97 family (for example, 97B1_BACFR and 97B2_BACTH) after two or three iterations. However, a statistically significant sequence similarity of GH97 family proteins with α-galactosidases is restricted to a fragment of about 100–150 amino acid residues (Figure 2). This fragment corresponds to the N-terminal half of the catalytic (β/α)_8_-barrel domain of glycosidases from the α-galactosidase superfamily [50-54]. This half of the domain is known to be more conserved than the C-terminal half [26]. Therefore, we can assume that the catalytic domain of the GH97 family proteins also has a similar (β/α)_8_-barrel fold.

In order to check whether the whole (β/α)_8_-barrel domain is present in GH97 family proteins, we tried to reconstruct their secondary and tertiary structure. The SWISS-MODEL program failed to unambiguously predict the type of the tertiary structure. The 3D-PSSM, GOR IV, and nnpredict programs were used for prediction of the protein secondary structure. The results obtained suggest that the central part of the GH97 family protein sequences represents a typical and complete (β/α)_8_-barrel domain (Figure 2). The N- and C-terminal parts of the sequences, mainly consisting of β-strands, most probably form two additional non-catalytic domains with an unknown function. However, different programs produce contradictory results regarding the number and exact location of the β-strands (data not shown). The non-catalytic domains of glycosidases from the α-galactosidase and α-glucosidase superfamilies are also predominantly composed of β-strands. At least some of these domains are involved in oligomerization and carbohydrate binding [2,54].

3D-PSSM searches of the PDB database with several GH97 family proteins used as a query sequence yielded the highest level of similarity with the GH27 family glycosidases [PDB: 1KTB, 1R46, and 1UAS]. Among other best hits we have found representatives of several other (β/α)_8_-barrel fold glycoside hydrolase families: GH2 (clan GH-A), GH5 (GH-A), GH13 (GH-H), GH17 (GH-A), GH18 (GH-K), and GH20 (GH-K), as well as some other enzymes with (β/α)_8_-barrel fold, for example *Bacillus subtilis *inositol utilization protein IolI [PDB: 1I6N]. These results are in agreement with the hypothesis about common origin of all (β/α)_8_-barrel protein domains, that evolved from an ancestral (β/α)_4 _half-barrel by a tandem gene duplication followed by a fusion [55-60].

In all known glycosidases with the (β/α)_8_-barrel fold, the amino acid residues involved in the active center are located on the C-termini of the β-strands [61], a similar location of the active site was found in many other (β/α)_8_-barrel fold enzymes [60]. It is well known that two acidic groups (Asp and/or Glu) are almost always involved in the glycosidase active center, playing the roles of nucleophile and proton donor [4-6]. Their sequence location has been determined for several representatives of the GH27 and GH31 families [54,62-69].

The Asp residue, playing the role of nucleophile, is located on the C-terminus of the fourth β-strand of the barrel. This residue is highly conserved among proteins of the α-galactosidase superfamily [23,26]. The homologous residue in the GH97 family proteins is more variable, being Asp in all members of three subfamilies (97b, 97c, and 97d) and Gly in the other proteins (subfamilies 97a and 97e), including 97A1_BACTH and 97A1_TANFO (Figure 2). Since these two proteins display the α-glucosidase activity [29,30,70] we can conclude that a residue, set in another site, plays the role of nucleophile at least in some proteins of the GH97 family. It should be noted that we have found a residue on the C-terminus of the fifth β-strand in GH97 family sequences that is Gly in 97b, 97c, and 97d subfamilies, but Glu and Asp in subfamilies 97a and 97e respectively (Figure 2). Therefore, this residue can be suggested as a possible nucleophile in glycosidases of 97a and 97e subfamilies. As a rule, the catalytically essential residues are highly conserved among enzymatically active members of a glycoside hydrolase family, being either Asp, or Glu. The distance between the carboxylic groups of the nucleophile and the proton donor should be similar in order to keep the catalytic machinery. Thus, the difference in the predicted nucleophile residue between 97a and 97e subfamilies is unexpected. However, this does not exclude the existence of a glycosidase activity in proteins with Asp residue at the fifth β-strand (subfamily 97e). To illustrate, in the GH32 family the Asp residue was experimentally shown to be the nucleophile, while several proteins of this family have Glu residue at the homologous position and at least some of them are catalytically active [10,11].

The proton donor of families GH27 and GH31 is located on the C-terminus of the sixth β-strand of the (β/α)_8_-barrel domain. It is outside of the N-terminal half of barrel, which can be unambiguously aligned with proteins of the GH97 family. However, on the C-terminus of the sixth β-strand of the predicted (β/α)_8_-barrel of the GH97 family there is an Asp residue, which is highly conserved in all subfamilies of the family (Figure 2). We suggest this residue as a possible proton donor. Taking into account another structure of the active center and significant sequence similarity of only a half of the catalytic domain, the current data do not support an inclusion of the GH97 family into the α-galactosidase superfamily.

As far as we know, 97A1_BACTH and 97A1_TANFO are the only enzymatically-characterized proteins in the GH97 family [2]. All other members of this family have been found recently during genome projects and are encoded by ORFs. Genes of this family are represented only in a limited number of Eubacteria from phyla Actinobacteria (1 genus), Bacteroidetes (4 genera), Planctomycetes (1 genus), and Proteobacteria (3 and 4 genera from α- and γ-classes, respectively), as well as in a unique Archaea (*Haloarcula marismortui*). However, many of these bacteria have several paralogous genes. The most interesting case is that of *B. thetaiotaomicron *ATCC29148, which has α-glucosidase SusB (97A1_BACTH) and 9 putative paralogues representing four GH97 subfamilies (Table I), at least two of the paralogues (97C1_BACTH and 97C2_BACTH) are also expressed *in vivo *[28]. This human commensal microorganism is known as a bacterium with the highest number of glycosidase and glycosyltransferase genes [27,71]. Taken together, these facts we can suggest that evolution of GH97 family proteins has been associated with multiple duplications, gene elimination, and horizontal transfer.

## Conclusion

The results of the sequence analysis allow us to distinguish five subfamilies in the GH97 family of glycoside hydrolases. The experimental data on the enzymatic activity are available only for two representatives of the GH97 family: α-glucosidases 97A1_BACTH and 97A1_TANFO [29,30,70]. However, we suppose that the other members of this family may also possess some glycosidase activities. Our data suggest that proteins of this family have a common evolutionary origin with glycosidases of the α-galactosidase superfamily. Many genes, encoding proteins of the GH97 family, are located in clusters with genes of glycoside hydrolases and other carbohydrate-active enzymes. For example, 97C1_BACTH and 97C2_BACTH (subfamily 97c) are encoded by genes of *B. thetaiotaomicron *located at a hemicellulose utilization locus together with eight other glycosidase genes (Figure 1). Taken together, these data support a recent suggestion to consider family GH97 (or GHX) as a new family of glycoside hydrolases [2,24]. The evolutionary relationship of GH97 proteins with glycosidases of the GH-D, GH-H, and GH-K (and probably GH-A) clans allows to extrapolate their common most important characteristics to glycoside hydrolases of the GH97 family. We can predict a similar (β/α)_8_-barrel fold of the catalytic domain and retaining mechanism of the glycoside bond hydrolysis for glycosidases of the GH97 family.

## Methods

Protein and nucleic sequences were retrieved from the NCBI database [72]. All proteins analyzed in this work were designated by a ten-letter name (see Table I). The search for homologous proteins was done using the PSI-BLAST [73] and Genomic BLAST at the NCBI server. The statistical significance threshold for including a sequence in the model (*E*-value) used by PSI-BLAST in the next iteration was either 10^-2 ^or 10^-3^, BLOSUM45 was used as a substitution matrix. Multiple sequence alignment was prepared manually using the program BioEdit [74] on the basis of BLAST pairwise alignments.

The multiple sequence alignment was used to implement classical phylogenetic inference programs, using either maximum parsimony or distance methods. Programs PROTPARS and NEIGHBOR from the PHYLIP package (version 3.6; [75]) were used. Moreover, programs SEQBOOT, PROTPARS, and CONSENSE and programs SEQBOOT, PROTDIST, NEIGHBOR, and CONSENSE were successively used to derive confidence limits, estimated by 1000 bootstrap replicates, for each node in the maximum parsimony and distance tree, respectively. The program TreeView Win32 (version 1.6.6; [76]) was used for drawing the trees.

An analysis of the order of the display sequence during searches by PSI-BLAST [73] was used for a preliminary division of a family into subfamilies. The latter was defined as a group of proteins that are displayed at the top of the list in a PSI-BLAST query results. Depending on particular criteria of the protein similarity used, the algorithm can split a family into a larger or smaller number of groups of proteins. Like in some of our previous works [10,23,24,77], in this study we define a subfamily as a group of proteins that have at least 30% sequence identity. Phylogenetic analysis was used in order to verify the obtained subfamilies and to clarify their boundaries. The monophyletic status was used as a criterion for the final definition of a subfamily.

The SWISS-MODEL modeling server [78] was used to predict the tertiary structure of proteins based on their amino acid sequences. The 3D-PSSM [79], GOR IV [80] and nnpredict [81] programs were used for prediction of the protein secondary structure. The 3D-PSSM program also was used to search the PDB database.

## Added in proof

After submission of the manuscript, six new sequences of GH97 family proteins have been deposited at the NCBI database. Five of them (97A1_SHEBA, 97A1_SHEFR, 97A1_SHEDE, 97A1_SHEAM, and 97A1_SPHAL) belong to subfamily 97a (Table I). The sixth protein 97X1_SOLUS cannot be unambiguously classified into any subfamily of the GH97 family on the basis of pairwise sequence comparison, composition of the signature sequence positions, and phylogenetic analysis. Most probably it corresponds to a new subfamily.
